# Experimental evidence for extra proton exchange in ribulose 1,5-bisphosphate carboxylase/oxygenase catalysis

**DOI:** 10.1080/19420889.2022.2039431

**Published:** 2022-02-15

**Authors:** Camille Bathellier, Guillaume Tcherkez

**Affiliations:** a ISARA, Department of Agroecology & Environment, Agrapole, Lyon, France; b Research School of Biology, Australian National University, Canberra, Australia; c Institut de Recherche en Horticulture et Semences, Université d’Angers, INRAe, Beaucouzé, France

**Keywords:** Rubisco, catalysis, isotope, kinetics, carboxylation, oxygenation

## Abstract

Despite considerable advances in the past 50 y, the mechanism of ribulose 1,5-bisphosphate carboxylase/oxygenase (Rubisco) catalysis is still not well understood. In particular, the movement and exchange of protons within the active site is not well documented: typically, kinetics of H exchange during the first steps of catalysis, i.e. abstraction of the H3 atom of ribulose 1,5-bisphosphate (RuBP) and enolization, are not clearly established. Here, we took advantage of reaction assays run in heavy water (^2^H_2_O) to monitor the appearance of deuterated RuBP and deuterated products (3-phosphoglycerate and 2-phosphoglycolate) with exact mass LC-MS. Enolization was reversible such that de-enolization generated not only monodeuterated RuBP (^2^H-[H-3]-ribulose 1,5-bisphosphate) but also dideuterated RuBP (^2^H_2_-[H-3,O-3]-ribulose 1,5-bisphosphate). Carboxylation yielded about one half deuterated 3-phosphoglycerate (^2^H-[H-2]-3-phosphoglycerate) and also a small proportion of dideuterated 3-phosphoglycerate (^2^H_2_-[H-2,O-2]-3-phosphoglycerate). Oxygenation generated a small amount of monodeuterated, but no dideuterated, products. (Di)deuterated isotopologue abundance depended negatively on gas concentration. We conclude that in addition to the first step of proton exchange at H3 occurring before gas addition (and thus influenced by the competition between de-enolization and gas addition), there is another proton exchange step between solvent water, active site residues, and the 2,3-enediol(ate) leading to deuterated OH groups in products.

## Introduction

**Figure 1.**
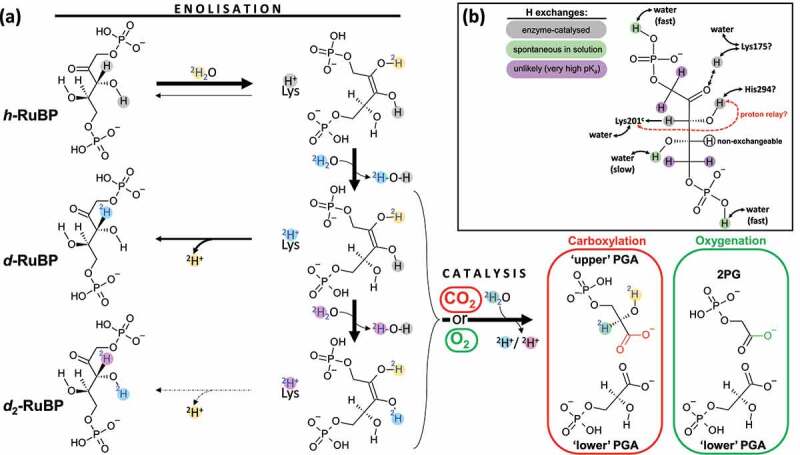
**Formal scheme showing proton exchanges in Rubisco catalysis**. (a) Chemical H exchanges during catalytic processes. The starting substrate is protiated ribulose 1,5-bisphosphate (denoted as *h*-RuBP). Enolization reversibility can generate deuterated RuBP, with a ^2^H atom at C3 (*d*-RuBP) or both C3 and O3 (*d*_2_-RuBP) via one or two solvent exchange steps. Solvent heavy water molecules involved in different steps of catalysis are labeled with different colors to allow tracing the fate of deuterons (^2^H^+^). For clarity, only one residue is shown in this figure, Lys 201 (numbering in spinach), which is responsible for proton abstraction in enolization in its carbamylated form. Note that the C3 proton abstracted during enolization is eventually lost during catalysis and reprotonation uses a solvent water molecule, thus forming a 3-phosphoglycerate (PGA) molecule (referred to as “upper”) deuterated at C2. In this panel, hydroxyl groups are simply represented as O–H for simplicity while in the chemical mechanism, H atoms can be shared with basic groups of active site residues, forming a partially charged oxygen atom. Note that the H (^2^H) atom at O2 in PGA can be slowly exchanged with solvent water after having been released by the enzyme (discussed in main text). (b) Summary of H exchange history of protons in RuBP.

Ribulose 1,5-bisphosphate carboxylase/oxygenase (Rubisco) is the fundamental enzyme of photosynthesis and catalyses carboxylation (CO_2_ addition) or oxygenation (O_2_ addition) of D-ribulose-1,5-bisphosphate (RuBP) and subsequent carbon–carbon cleavage to form two molecules of 3-phospho-D-glycerate (PGA) (with CO_2_) or one molecule of PGA plus one molecule of 2-phospho-glycolate (with O_2_) (Figure 1(a)). The reaction proceeds through several elemental steps including enolization, yielding a 2,3-enediolate which is the substrate of gas addition [1,2]. Despite the importance of this reaction, which abstracts about 120·10^9^ tons of carbon from the atmosphere each year, events in catalysis remain unclear, in particular how protons are transferred between residues and exchanged with the solvent. Enolization, which is believed to be partially rate-limiting [3,4], involves both proton abstraction at H3 (proton attached to C3) and protonation at O2 (negatively charged oxygen atom attached to C2) (Figure 1(a,b)). Proton abstraction involves carbamylated Lys 201 (numbering in spinach; carbamylated Lys 201 is denoted thereafter as Lys 201^c^) [5,6]. The origin and the fate of the proton given to O2 is unclear. Cleland and coworkers [2] have suggested that the H3 proton abstracted by Lys 201^c^ could shuttle to O2 and then to Lys 175 and may eventually undergo proton exchange with the solvent. However, the role of Lys 175 in enolization remains unclear since it could be either a proton acceptor or a proton donor and play a role at later steps in PGA formation [2,5,7–9]. Also, other residues (Thr 175, Lys 177 and His 294) can be involved in protonation-deprotonation events. In fact, the protein structure at 1.5 Å and 1.85 Å resolution with Mg^2+^ and 2-carboxy-D-arabinitol 1,5-bisphosphate (carboxylation transition state analogue) as a ligand suggests that O2 is close to Lys 175 and Thr 173, while O3 is close to His 294 and Lys 201^c^ and the carboxylate is at a short distance to Lys 177 [10,11].

The role of water molecules in catalysis also seems to be rather complicated [reviewed in [12]]. The structure of the protein crystallized with Ca^2+^ shows that a water molecule participates in the Mg^2+^ coordination sphere [13] and could participate in the reaction. However, water is believed to be mostly excluded from the chemistry of CO_2_ addition and cleavage of the six-carbon intermediate. In fact, the binding of RuBP appears to sequester Lys 201^c^ from the bulk and no water molecule is seen near the carbamate, implying the latter residue experiences a substantially non-aqueous environment [6,14]. Also, structural analysis as shown that both gaseous substrates CO_2_ and O_2_ are embedded in a positively charged cavity located at the C-terminal end of the β-barrel of the catalytic domain and closed with a negatively charged lid formed by the N-terminal domain of an adjacent L subunit [15]. Labeling experiments with tritiated RuBP ([^3^H-3]-RuBP) and spinach Rubisco showed negligible labeling in PGA [16] and, therefore, the H3 proton is believed to be lost before the production of upper PGA. By contrast, enolization involves protons from water, as shown by pioneering assays of isotopic exchange between the H3 proton and solvent water: with bacterial Rubisco (*Rhodospirillum rubrum*) assayed in tritiated water (^3^H_2_O) with protiated RuBP, RuBP molecules not consumed by the reaction are eventually fully ^3^H-labeled [3]. Similarly, plant Rubiscos assayed in heavy water (^2^H_2_O) yield deuterated RuBP [17,18], allowing calculation of the reverse commitment to catalysis (de-enolization to gas addition ratio) of about 7 (carboxylation) and 500 (oxygenation) μM [17]. Interestingly, in ^2^H_2_O, there is an enhancement of the production of side product pyruvate, which comes from β-elimination (competing with PGA production) of the six carbon intermediate [19]. That is, with a deuterium atom, reprotonation (redeuteration) of C2 is relatively slow, thereby favoring wasteful cleavage of the carbon-phosphate bond. This shows that the proton used for reprotonation may have exchanged with the solvent at some point before or during gas addition, but underlying specific chemical events are unknown. A better knowledge of the chemical path followed by protons during Rubisco catalysis is therefore needed. Here, we took advantage of assays with Rubisco from tobacco (*Nicotiana tabacum*), natural (protiated) RuBP and heavy water (98% ^2^H_2_O) as a solvent, and followed isotopologues of substrate RuBP and products using high-resolution (exact mass) LC-MS.

## Results and discussion

### Liberation of deuterated RuBP via de-enolization

**Figure 2.**
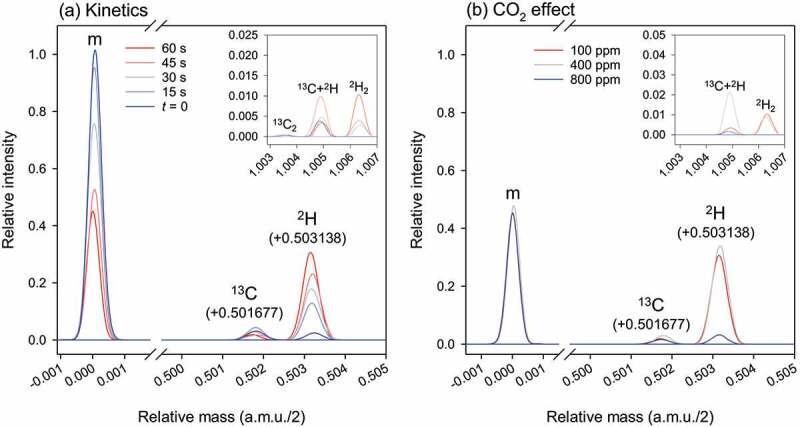
**RuBP isotopologues observed during Rubisco-catalyzed carboxylation**, monitored using high resolution (exact mass) LC-MS. Substrate ribulose 1,5-bisphosphate (RuBP) was natural (protiated RuBP) and the solvent was heavy water (^2^H_2_O). Here, signals associated with the major ion are shown, i.e., [M–2H]^2 –^ with a monoisotopic *m/z* value of 153.984921 a.m.u. Units of the *x*-axis are in half a.m.u. because of the charge (–2) of the ion of interest. (a) Kinetics of protiated ^12^C (monoisotopic, denoted as m) and protiated ^13^C RuBP disappearance and formation of deuterated RuBP (^2^H) during the reaction (assay of 60 seconds with CO_2_ equilibrated at 100 ppm in the gas phase). The inset shows the appearance of ^12^C dideuterated and ^13^C monodeuterated RuBP. (b) Isotopologue composition at 60s when CO_2_ is equilibrated at 100, 400 or 800 ppm in the gas phase. In all cases, the initial amount of RuBP was adjusted to reach ≈40% of initial concentration at the end of the assay. Note the much higher content in deuterated RuBP at low (100 ppm) and moderate (400 ppm) CO_2_ concentration, compared to high CO_2_ (800 ppm). As in (a), the inset shows ^12^C dideuterated and ^13^C monodeuterated isotopologues.

When the carboxylation assay was run in ^2^H_2_O as a solvent with protiated RuBP as a substrate, there was a progressive monodeuteration in RuBP (+0.503138 a.m.u. for the *m/z* feature of interest). This deuteration reflected the formation of ^2^H-3-RuBP (*d*-RuBP) via de-enolization (Figure 2(a)). Under our conditions, about 40% of RuBP appeared to be monodeuterated while total RuBP declined since it was consumed by the reaction (Figure 2(a)). When dissolved CO_2_ concentration was increased to 24 µM (800 ppm in equilibrating gas phase) and RuBP initial concentration increased to keep the same proportion of consumed RuBP, there was a strong decline in the appearance of *d*-RuBP (Figure 2(b), blue), demonstrating that de-enolization directly competes with gas addition. Importantly, we also found the dideuterated RuBP isotopologue (*d*_2_-RuBP, +1.006277 a.m.u.). Its content, which was always very small, increased with time during the assay and was undetectable with no reaction. This indicates that a second proton exchange occurs and involves the enzyme. It is likely that in dideuterated RuBP, one deuterium atom is at H3 and the second deuterium atom is carried by an hydroxyl (OH) group (see H inventory in RuBP summarized in Figure 1(b)). The two O–H bonds that are known to be weakened during the reaction are at O2 and O3 of the 2,3-enediol(ate) intermediate. However, only the H atom at O3 is retained in RuBP (since RuBP has a C=O group at C2). Secondary H exchange at other sites (such as C1) are less likely considering their pK_a_ values [20,21]. Therefore, it is plausible that *d*_2_-RuBP corresponds actually to ^2^H_2_-[H-3,O-3]-ribulose 1,5-bisphosphate. The ^2^H atom carried by O3 comes from water either directly or via an active site residue if there is a proton relay between water, Lys residue(s) and H at O3.

### Production of deuterated products

**Figure 3.**
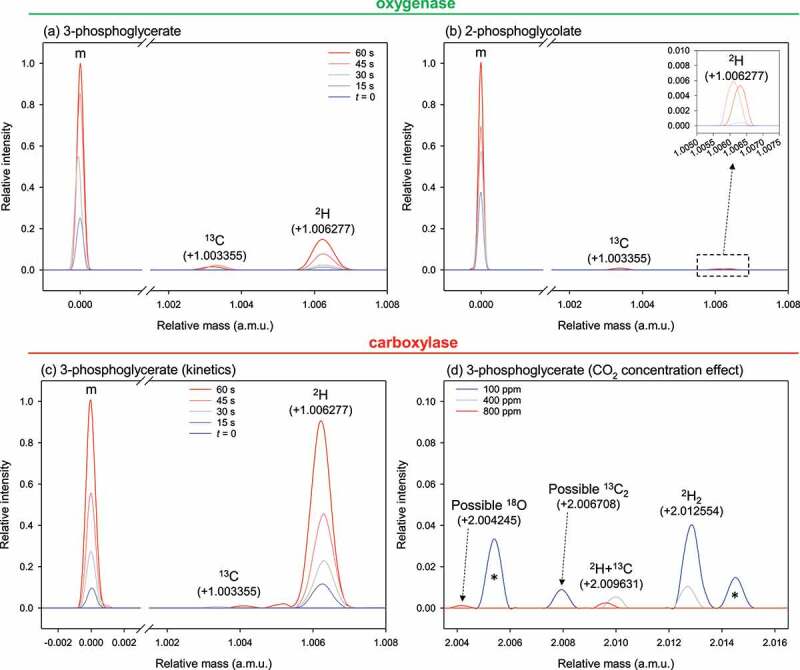
**Isotopologues of products of Rubisco catalysis**: oxygenation (a,b) and carboxylation (c,d), monitored using high-resolution (exact mass) LC-MS analysis. Substrate ribulose 1,5-bisphosphate (RuBP) was natural (protiated RuBP) and the solvent was heavy water (^2^H_2_O). Major ions of 2-phosphoglycolate and 3-phosphoglycerate are (monoisotopic): [M–H] ^–^ at 154.9745 a.m.u. and [M–H] ^–^ at 184.9851 a.m.u., respectively. As in Figure 2, the figure shows the monoisotopic species (m), ^13^C and ^2^H isotopologues (a,b,c) and double isotopologues (inset of b, and d). Results presented here are associated with a 60s-assay (a,b,c) or the effect of CO_2_ concentration (d) equilibrated with 100, 400 or 800 ppm in the gas phase. In (d), the asterisk (*) stands for other features (*m/z*) present in the assay and unrelated to 2-phosphoglycolate or 3-phosphoglycerate. In (a) and (b), no dideuterated 3-phosphoglycerate or 2-phosphoglycolate could be observed.

Isotopic species of products was also monitored with exact mass LC-MS (Figure 3). As expected, there was an increase in both PGA and 2PG during the oxygenase assay. Interestingly, monodeuterated PGA and 2PG also accumulated (at +1.006277 a.m.u.) during the reaction. Since there is no reprotonation to form ‘lower’ PGA during the oxygenase reaction, this shows that deuterated species came from isotopic exchange within the active site. Such isotopic exchange probably occurred at C1, O4 and/or C5 (numbers refer to numbering in RuBP). It is also possible that deuterated PGA came from residual carboxylase activity (despite constant equilibration with CO_2_-free N_2_/O_2_ to keep oxygenation conditions) caused by remaining traces of bicarbonate used beforehand to pre-activate the enzyme (i.e. to carbamylate Lys 201). However, this cannot explain 2PG deuteration (Figure 3(b), inset), which must come from secondary isotopic exchange at C1. As expected, the carboxylase reaction generated monodeuterated PGA, coming from reprotonation with solvent water during catalysis (green ^2^H atom in Figure 1). Unlike the oxygenase assay, there were dideuterated molecules essentially detectable at low CO_2_ (Figure 3(d)). It is likely that the second deuterium atom was carried by an OH group rather than the C atom from C1 in RuBP, since the observed amount is proportionally much larger than that of the monodeuterated species of 2PG.

### Implications for catalysis

**Figure 4.**
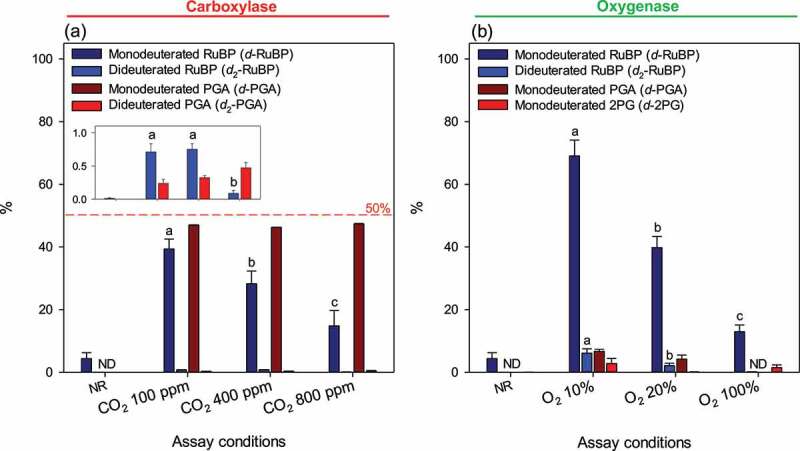
**Abundance of deuterated isotopologues in substrate and products of Rubisco catalysis**. Substrate ribulose 1,5-bisphosphate (RuBP) was natural (protiated RuBP) and the solvent was heavy water (^2^H_2_O). RuBP and 3-phosphoglycerate (PGA) after 60s of carboxylase assay (a), RuBP, PGA and 2-phopshoglycerate (2PG) after 60s of oxygenase assay (b). The inset in (a) is a magnification to facilitate reading the percentage of dideuterated species. The value shown here is the percentage (%) with respect to the total pool size of the metabolite of interest; for example, 40% *d*-RuBP means than monodeuterated RuBP represents 40% of total RuBP (total = non-deuterated + monodeuterated + dideuterated + other isotopic forms such as the ^13^C isotopologue). Data shown are mean ± SE (*n* = 3). Gases concentrations are shown with respect to the gas phase used to equilibrate the reacting medium. Letters stand for statistical classes (*P*< 0.05). ND, not detected. NR, no reaction.

Amounts of isotopic species of RuBP, PGA and 2PG formed during assays are compiled in Figure 4. Results are expressed in % of total content of the metabolite of interest. We recognize that whenever OH deuteration is involved, values shown here are probably slightly underestimated due to possible isotopic exchange (i.e., ^2^H loss) during sample processing for LC-MS analysis. Nevertheless, such spontaneous exchange is slow [22] and furthermore, there was a clear difference between samples with and without reaction, showing the role played by the enzyme. Interestingly, there was slightly less (≈48%) than 50% of monodeuterated PGA. This is explained by (*i*) the contribution of dideuterated PGA (less than 0.5%, Figure 4(a), inset) and (*ii*) the fact the assay was not run in 100%, but 98% ^2^H-water. In both oxygenase and carboxylase reactions, monodeuterated RuBP formation by de-enolization decreased with gas concentration. Monodeuterated PGA was not detected at high dissolved O_2_, suggesting that isotopic exchange at the OH group at C4 occurred prior to gas addition and/or that high O_2_ was required to prevent the enzyme from consuming traces of dissolved CO_2_ in the reaction medium. Monodeuterated 2PG was observed at all O_2_ concentrations, suggesting spontaneous isotopic exchange upon 2PG release from the active site. The content in dideuterated RuBP depended negatively on gas concentration (both CO_2_ and O_2_) showing that deuteration took place within the active site prior to gas addition.

The data presented here provides compelling evidence that Rubisco catalysis is accompanied by proton exchanges with the solvent at molecular sites other than C2/3 (abstraction at C3 during RuBP enolization and reprotonation at C2 in PGA). To our knowledge, the present results provide the first evidence that there is a proton exchange other than at H3, after the generation of the 2,3-enediol(ate) and before gas addition. The site concerned by this exchange is very likely a OH group prone to deprotonation-reprotonation (i.e., acid-base exchange with amino acid residues of the active site). In other words, *d*_2_-RuBP formed by de-enolization carries a deuteron at H3 and probably at O3. In fact, from consideration of crystal structures and mutation effects, the H atom bound to O3 is likely to be exchanged with His 294 [10,23]. It has been proposed that in the last steps of enolization, it can be replaced by the H3 atom previously abstracted by Lys 201^c^ in the first step of enolization (proton relay hypothesis; it is shown in Figure 1 whereby the gray proton at O3 is lost and replaced by the blue deuteron from Lys 201^c^) [2]. The proton relay hypothesis is also supported by the fact that the use of monodeuterated RuBP as a substrate (^2^H-3-ribulose 1,5-bisphosphate) affects the ^12^C/^13^C kinetic isotope effect during carboxylation [17]. It means that bonding in the reaction intermediate and/or the transition state involves somehow the abstracted H3 proton before it is washed out to the solvent. The most likely is that the H3 proton interacts with O2 and/or O3 in the 2,3-enediol(ate) because it is sufficiently close to the C atom attacked by CO_2_ in carboxylation and therefore, it can affect significantly the ^12^C/^13^C kinetic isotope effect.

The involvement of several proton exchanges during enolization and before gas addition is thus of importance for catalysis since it has potential effects on gas addition itself. That is, there is an interdependence of reaction steps during catalysis. It further suggests that in terms of enzyme evolution, the adaptation of the active site to adjust kinetic properties when CO_2_ and O_2_ availability varies, should not only involve residues directly involved in CO_2_ attack but also H bonding during enolization. This conclusion is in line with the recognized catalytic trade-off during Rubisco catalysis suggested by the comparison of kinetic properties in different Rubisco forms [24].

## Methods

### Rubisco purification and assays

Tobacco (*Nicotiana tabacum* var. Wisconsin) Rubisco was purified after [25] by precipitation from tobacco leaf extracts using 12% PEG, and then crystallization in Tris (25 mM) at pH 7.2. Rubisco crystals were collected, washed and redissolved (in Tris 5 mM pH 7.6, 20% glycerol) for storage at – 80°C. Rubisco assays were conducted as in [17]. Assays were carried out at 25°C in septum-capped 2-mL vials filled with 300 µL buffer (HEPES 100 mM, 20 mM MgCl_2_, pH 8). The reaction medium was first equilibrated for 1 h with a gas mixture CO_2_/O_2_ in N_2_ produced with high precision mass-flow controllers (FC260, Tylan). CO_2_ and O_2_ mole fractions were continuously monitored with an IRGA (Li-6251, Li-Cor) and an oxygen sensor (MAX-250, Maxtec). 10 µL of RuBP solution was then injected to get a final concentration of about 3 mM (adjusted to have about 40% consumption after reaction). The reaction was started with 10 µL of Rubisco extract that had been pre-activated for 20 min with 20 mM NaHCO_3_ and 15 mM MgCl_2_. The reaction ran under constant gas bubbling and was quenched with 240 µL EDTA at 100 mM followed by snap-freezing in liquid nitrogen and lyophilization (freeze-dried).

### LC-MS analyses

Isotopologue composition of RuBP and products was assessed using high-resolution LC-MS Orbitrap Q Exactive (Thermo Scientific). Lyophilized samples from assays were redissolved in water, ions were removed with washed Dowex 50 H+ and the eluate was instant-frozen, lyophilized, and redissolved in 100 µL water. Samples were injected directly (i.e. by infusion). MS analysis was operated in negative polarity in the full MS scan mode (mass scan range 50–750 *m/z*) with the following source settings: source voltage 3,500 V, resolution 70,000, AGC target 1 · 10^6^, mass scan range 60–600 m/z, sheath gas 40, auxiliary gas 10, sweep gas 1.5, probe temperature 300°C, capillary temperature 250°C and S-lens RF level 50.
